# Prevalence of Estrogen Receptor Alpha (*ESR1*) Somatic Mutations in Breast Cancer

**DOI:** 10.1093/jncics/pkac060

**Published:** 2022-08-12

**Authors:** Connor J Kinslow, Ashley Tang, Kunal R Chaudhary, Simon K Cheng

**Affiliations:** Department of Radiation Oncology, Vagelos College of Physicians and Surgeons, Columbia University Irving Medical Center, New York, NY, USA; Department of Radiation Oncology, Vagelos College of Physicians and Surgeons, Columbia University Irving Medical Center, New York, NY, USA; Department of Radiation Oncology, Vagelos College of Physicians and Surgeons, Columbia University Irving Medical Center, New York, NY, USA; Department of Radiation Oncology, Vagelos College of Physicians and Surgeons, Columbia University Irving Medical Center, New York, NY, USA

## Abstract

Estrogen receptor–positive breast tumors, which initially respond effectively to endocrine therapy, progress due to acquired endocrine therapy resistance, including genomic alterations in estrogen receptor alpha (*ESR1*). A recent study has suggested that there is a sufficient number of preexisting *ESR1* mutations acting as an intrinsic resistance mechanism to warrant primary screening. We determined the incidence of de novo *ESR1* mutations in hormone-positive treatment-naïve primary breast tumors using 12 publicly available international datasets in the cBioPortal. The prevalence of mutation was statistically significantly lower in treatment-naïve primary tumors (n* *=* *6 of 3682, 0.16%) than in metastatic (n* *=* *156 of 1089, 14.3%, 2-sided *P *<* *.001) or previously treated primary tumors (n* *=* *11 of 92, 12.0%, 2-sided *P *<* *.001). Pathogenic *ESR1* mutations are a common mechanism of acquired but not intrinsic resistance to endocrine therapy and may not warrant universal testing of primary breast cancer populations.

Approximately 70%-80% of breast cancers are estrogen receptor (ER) positive, which respond effectively to endocrine therapy. However, breast tumors have been shown to progress due to acquired endocrine therapy resistance, including genomic alterations in estrogen receptor alpha (*ESR1*) (1). *ESR1* mutations are common in metastatic and endocrine-resistant disease and are associated with a more aggressive clinical course. Recently, Dahlgren et al. (2) also showed that pathogenic mutations in *ESR1* may be a biomarker of intrinsic resistance to endocrine therapy in primary breast cancer patients. The authors analyzed 3217 patients enrolled in the multicenter Sweden Cancerome Analysis Network - Breast (SCAN-B) Initiative, which is the largest prospective population-based cohort of primary breast tumor patients undergoing routine RNA sequencing. *ESR1* pathogenic mutations occurred in 1.1% of ER-positive tumors and were associated with poorer relapse-free and overall survival after endocrine therapy. Although *ESR1* mutations were recently described as a mechanism of acquired resistance to endocrine therapy, this is the first study, to our knowledge, to identify preexisting *ESR1* mutations as a biomarker of intrinsic resistance in the adjuvant setting. The authors conclude that if their results are replicated, *ESR1* screening should be considered in ER-positive primary breast cancer. We therefore sought to validate their results using publicly available international datasets in the cBioPortal (3).

All publicly available breast and pan-cancer databases in cBioPortal were queried for clinically and genomically annotated data. Primary and metastatic breast tumor samples were included in our analysis if information on hormone status was available. Putative driver (pathogenic) mutations versus variants of unknown significance were defined via OncoKB and Cancer Hotspots annotations. Treatment status was included when available. The prevalence of *ESR1* mutations was compared between groups using Fisher’s exact test. Continuous variables were compared by the Wilcoxon test. Tests of statistical significance were 2-sided, and *P* values less than .05 were considered statistically significant. Statistical analyses were conducted using the cBioPortal website and RStudio Version 1.4.1106 (RStudio, Inc) software.

We surveyed 7103 primary or metastatic breast tumors from 6882 patients in 12 studies, including 4863 hormone-positive samples derived from 4727 patients (Table 1) (4-13). In the Metastatic Breast Cancer Project (7), pathogenic *ESR1* mutations were more common in hormone-positive metastatic versus primary tumors (13.2% vs 2.5% [5 of 38 vs 4 of 161], *P *=* *.01) and pretreated versus treatment-naïve tumors (11.1% vs 0% [4 of 36 vs 0 of 80], *P *=* *.008), consistent with previous reports (4,13). Additionally, median times from diagnosis to sample collection and metastatic diagnosis were longer in *ESR1* mutant tumors (100 vs 1 months, *P *=* *.001 and 68 vs 2 months, *P *=* *.04, respectively), suggesting a dose-response relationship between endocrine therapy exposure and the development of *ESR1* pathogenic mutations.

**Table 1.** Frequency of pathogenic *ESR1* mutations in hormone-positive tumors^a^

Study	Description	No. of patients	Age < 50, %	HER2+, %	Grade 1–2, %	Stage I-II, %	No. of samples	Variant allele frequency threshold	*ESR1*-mutant, No.	*ESR1*-mutant, %
Primary breast cancer, treatment-naïve										
MBC project, *ESMO Open*, 2018 (7)	United States	63	73.0	27.6	66.7	32.3	69	0.01	0	0
MSKCC,^l^*Cancer Cell*, 2018 (4)	United States, prospective	739	38.6	7.6	51.1	69.7	756	0.02	4	0.53
TCGA, *Cell*, 2018 (6)	International, retrospective/prospective	659	29.1	30.1	—	73.1	659	0.03	2	0.30
METABRIC, *Nature*, 2012 (9)	United Kingdom/Canada^b^	1844	17.8	7.4	61.0	92.5	1844	None^g^	0	0
SMC, *Nature Comm*, 2018 (12)	Korea, prospective^c^	131	87.0	21.4	—	66.4	131	—	0	0
Broad, *Nature*, 2012 (8)	Vietnam/Mexico	58	51.7	16.1	74.3	81.0	58	Other^h^	0	0
Sanger, *Nature*, 2012 (11)	United Kingdom^b^	81	39.5	30.9	56.8	—	81	—	0	0
CPTAC, *Cell*, 2020 (5)	International, prospective	83	22.9	9.0	—	66.7	83	0.01	0	0
Total		3659	28.6	13.5	58.6	79.5	3682		6^i^	0.16
Metastatic breast cancer										
MBCproject, *ESMO Open*, 2018 (7)	United States	32	85.0	35.5	52.6	33.3	38	0.01	5	13.6
MSKCC,^l^*Cancer Cell*, 2018 (4)	United States, prospective^d^	788	53.5	11.9	28.8	51.7	877	0.02	132	15.1
INSERM, *PLoS Med*, 2016 (13)	France, prospective^e^	143	—	0.0	—	—	143	0.1	16	11.2
China Pan-cancer (3)	China^f^	31	51.6	—	—	—	31	—	3	9.7
Total		994	54.2	12.9	29.5	51.3	1089		156^j^	14.3
Primary breast cancer, previously-treated										
MBCproject, *ESMO Open*, 2018 (7)	United States	27	44.4	18.5	58.3	24.0	28	0.01	2	7.1
MSKCC,^l^*Cancer Cell*, 2018 (4)	United States, prospective	47	53.2	9.1	55.9	10.6	47	0.02	9	19.1
China Pan-cancer (3)	China	17	70.6	—	—	6.7	17	—	0	0
Total		91	53.8	12.7	56.9	14.8	92		11^k^	12.0

a All datasets are publicly available on cBioportal. Hormone-positive samples were identified from 7103 samples from 6882 patients in 12 studies. The MET500 (*Nature*, 2017) and ICGC (*Nature*, 2020) datasets lacked information on hormone status and were excluded.

b Treatment status not specified.

c Patients were 88% premenopausal and 95% treatment naïve.

d Patients received a median of 3 previous lines of therapy (range 1–15).

e Patients pooled from genomic screening for SAFIR01 (*Lancet Oncol.*, 2014), SAFIR02 (*Nature Med.*, 2021), SHIVA (*Lancet Oncol.*, 2015), and MOSCATO (*Cancer Discovery*, 2014) prospective trials. A total 84% received prior endocrine therapy.

f All patients received previous endocrine therapy.

g No threshold was used for mutations that were present/confirmed in the COSMIC database. Variants were removed if present in the normal tissue pool.

h Variants were included if statistically above noise and not present in normal tissue pool.

i Mutations identified in exons 380 (n* *=* *3), 536 (n* *=* *1), 537 (n* *=* *1), and 538 (n* *=* *1).

j Mutations identified in exons 380 (n* *=* *22), 422 (deletion, n* *=* *3), 463 (n* *=* *1), 536 (n* *=* *8), 537 (n* *=* *66), and 538 (n* *=* *63).

k Mutations identified in exons 380 (n* *=* *2), 463 (n* *=* *1), 536 (n* *=* *1), 537 (n* *=* *5), and 538 (n* *=* *2).

l Memorial Sloan-Kettering Cancer Center.

Across all studies, there were 6 tumors with pathogenic *ESR1* mutations identified among 3682 hormone-positive treatment-naïve primary breast tumors. The prevalence of mutation was statistically significantly lower in treatment-naïve primary tumors (n* *=* *6, 0.16%) than in metastatic (n* *=* *156 of 1089, 14.3%, *P *<* *.001) or previously treated primary tumors (n* *=* *11 of 92, 12.0%, *P *<* *.001). Of the 4 hormone-positive treatment-naïve primary breast tumors with *ESR1* mutations identified in the MSKCC dataset, follow-up times were shorter than 7 months and there were no progressions, distant recurrences, or deaths, precluding meaningful interrogation of the prognostic impact of preexisting *ESR1* mutations. Two additional patients with *ESR1* mutant tumors in the TCGA database were followed-up for 13 and 50 months, respectively, without progressions, recurrences, or deaths. Two patients in the China Pan-cancer dataset with *ESR1* mutant treatment-naïve primary tumors were excluded because of unknown hormone status. In a sensitivity analysis, including these 2 tumors with unknown hormone status would increase the prevalence of *ESR1* mutations to 0.21% in hormone-positive treatment-naïve primary tumors.

These data provide evidence that pathogenic *ESR1* mutations are a common mechanism of acquired but not intrinsic resistance to endocrine therapy. Differences in prevalence may be attributed to sequencing methodology and vary within populations. Additional studies are necessary to determine if the incidence is sufficient to warrant universal testing of primary breast cancer populations.

## Funding

No funding was used for this study.

## Notes

**Role of the funder:** Not applicable.

**Disclosures:** SKC reports personal fees and non-financial support from AbbVie and Sanofi. The other authors do not have any disclosures.

**Author contributions:** CJK: conceptualization, data curation, formal analysis, investigation, methodology, writing—original draft, writing—review and editing. AT: writing—original draft, writing—review and editing. KRC: supervision, writing—review and editing. SKC: conceptualization, methodology, project administration, supervision, writing—original draft, writing—review and editing.

**Acknowledgements:** We thank the creators and contributors of the cBioPortal.

## Data Availability

Data is available as cited in the cBioPortal.
